# Predicting multiple long-term conditions with role limitation at age 46 using early-life data from the 1970 British Cohort Study

**DOI:** 10.1136/bmjph-2025-004443

**Published:** 2026-06-28

**Authors:** Kim Alipio, Sebastian Stannard, Nida Ziauddeen, Simon DS Fraser, Nisreen A Alwan

**Affiliations:** 1 University of Southampton Faculty of Medicine, Southampton, UK; 2 NIHR Applied Research Collaboration Wessex, Southampton, UK; 3 University of Southampton, NIHR Southampton Biomedical Research Centre, Southampton, UK; 4 School of Primary Care, University of Southampton, Southampton, UK

**Keywords:** Primary Prevention, Epidemiologic Research Design, Epidemiology, Public Health

## Abstract

**Background:**

There is minimal research investigating whether multiple long-term conditions (MLTCs) can be predicted using early-life data. We examined whether the risk of later-life MLTCs that impact everyday functioning can be predicted from factors recorded at birth and age 5.

**Methods:**

Data from 5007 participants aged 46 in the 1970 British Cohort Study were analysed. The outcome was two or more self-reported MLTCs with/without role limitation (ie, impacting everyday life functioning) due to a physical or mental health condition. Backwards stepwise logistic regression was used as a variable selection approach to identify early-life predictors that improved model discrimination for four models: (1) age 5 predictors only and MLTC with role limitation, (2) age 5 predictors only and MLTC without role limitation, (3) age 5 and birth predictors and MLTC with role limitation and (4) age 5 and birth predictors and MLTC without role limitation. Model discrimination was assessed using the area under the curve (AUC).

**Results:**

11.9% of the sample reported MLTCs with role limitation. The set of variables selected differed across models, reflecting differences in which predictors most improved predictive discrimination for each outcome. The highest AUC (0.66) was for model 3 including age 5 and birth predictors of MLTCs with role limitation at age 46. Retained age 5 predictors included the number of immunisations, sex, Rutter behaviour score, maternal malaise index (an indication of psychological distress), asthma diagnosis, if the child was seen by a doctor/nurse in school, whether the parent viewed their child as equals, and if the parent believed the child should have unquestioning obedience towards them. Retained birth predictors included: maternal smoking, gestational age, parity and if the mother ever had a teenage pregnancy.

**Conclusions:**

Using early-life birth cohort data from birth and age 5 did not predict MLTCs with role limitation at age 46 well. Exploration of predictors during other time points in childhood could better inform preventative interventions.

**WHAT IS ALREADY KNOWN ON THIS TOPIC**

- There is limited evidence on whether multiple long-term conditions (MLTC) can be predicted using information collected in early childhood.

**WHAT THIS STUDY ADDS**

- This study examined whether MLTC that affect daily functioning at age 46 can be predicted using national British birth cohort data collected at birth and at age 5.
- The strongest predictive model achieved an area under the curve value of 0.66, indicating relatively poor discriminatory ability.

**HOW THIS STUDY MIGHT AFFECT RESEARCH, PRACTICE OR POLICY**

- The findings suggest that prediction based solely on information from birth and early childhood is limited, indicating a need to explore further childhood, adolescent or adult time points to improve predictive models and guide preventative strategies.
- The study highlights that other developmental stages may provide more informative opportunities for preventing or mitigating midlife MLTC.

## Introduction

 Multiple long-term conditions (MLTCs), defined as living with two or more physical or mental health LTCs, have increased in prevalence globally over the last two decades, with a global prevalence now estimated at 37.2%.^1^ MLTCs can exert significant strain on both patients and health and social care systems, given that patients with MLTCs often need frequent healthcare visits, specialist appointments, diagnostic tests and multiple prescriptions.^2 3^ The UK’s Major Conditions Strategy estimates that patients with MLTCs account for over half of NHS costs, including around 50% of hospital admissions, outpatient visits and primary care consultations.^4 5^ However, MLTCs are no longer considered a condition characterised by older age, with most people living with MLTCs under 65 years.^6^ Earlier onset MLTCs often occur among individuals with higher socioeconomic deprivation, from certain ethnic groups and in women, thereby widening health inequalities.^7 8^

The increasing prevalence of MLTCs has supported a shift towards a need for a more complex understanding of MLTCs. For example, National Institute for Health and Care Excellence (NICE) guidance recommends considering ‘burden’ within the context of MLTCs,^9^ and a recent qualitative evidence synthesis of the experience of living with MLTCs identified the multifaceted nature of the impact of MLTCs on many aspects of life.^2^

Wider adult social factors, alongside biological and environmental factors, have been found to influence MLTCs throughout the life course,^10^ and there is research highlighting that early-life factors such as childhood illness, parental socioeconomic status, child maltreatment and educational attainment, are associated with single LTCs in adulthood.^11^,^13^ Despite this, minimal research investigates whether MLTCs can be predicted using data from early life, defined as the period from preconception until age 18. Identifying early-life predictors that are potentially causal is important for informing the design of effective preventative interventions, and targeting modifiable early exposures may help prevent or delay the onset of burdensome MLTCs, thereby reducing pressure on health and social care systems.

This study is framed within a life course framework, which suggests that experiences and exposures in early life can shape health trajectories across adulthood.^14 15^ These influences may operate through the accumulation of risk across multiple developmental stages or through critical or sensitive periods in which exposures have disproportionate and lasting effects on later health.^16 17^ Three of these critical periods, relevant to this paper, include preconception, prenatal, perinatal, infancy and early childhood.^16 17^ These periods are particularly important for the establishment of biological, behavioural and social pathways that underpin later health and functioning.^18^

A substantial body of life course research has demonstrated that childhood socioeconomic conditions, parental health behaviours, developmental indicators and early health status are associated with adult MLTCs.^16 17 19 20^ This evidence reflects what has been termed the ‘long arm of childhood’, whereby early advantages or disadvantages exert influence decades later.^16 17 19 20^ Supporting this, the Life Course Health Development framework conceptualises health as emerging from cumulative interactions between biological, behavioural, social and environmental processes across the life span, with childhood representing one of the most influential developmental stages.^14 21^

Within this context, examining whether early-life factors can predict later-life MLTCs is both theoretically grounded and empirically justified. Although prediction across a long temporal period is challenging, testing the extent to which early predictors retain predictive value provides important insights into the role of childhood factors in shaping long-term MLTCs trajectories. By using longitudinal cohort data with prospectively measured early-life exposures, this study evaluates whether the risk of later-life MLTCs can be predicted using data recorded at birth and age 5. In doing so, it contributes to understanding the extent and the limits of early-life predictability within long-term MLTC development.

There are three gaps in the literature that this paper addresses. First, although previous studies have explored associations between early-life factors and individual LTCs in adulthood, very little work has examined whether prospectively collected early-life data can predict the development of MLTCs rather than single conditions. Second, existing MLTC research rarely includes measures of burden, such as role limitation, despite their relevance for public health planning. As a result, it remains unclear whether early-life factors can predict not only the presence of MLTCs but also their functional impact. Third, no prior studies have systematically evaluated which early-life domains, previously conceptualised to influence long-term health trajectories,^22^ add meaningful predictive value when modelled together using a life course prediction approach.

## Methods

This work was conducted as part of a National Institute for Health and Care Research-funded research collaboration titled Multidisciplinary Ecosystem to study Lifecourse Determinants and Prevention of Early-onset Burdensome Multimorbidity.^23^

### Dataset

The 1970 British Cohort Study (BCS70) tracked 17 196 individuals born in 1 week in 1970 across England, Scotland and Wales.^24^ Since birth, data were gathered approximately every 4 years in a series of 11 data collection ‘sweeps’—four in childhood and seven in adulthood. The datasets produced and analysed in this study are held by the UK Data Service and are accessible under an End User Licence Agreement. Data were collected using a combination of self-reported and objective measurements. A user guide is available from the Centre for Longitudinal Studies and a cohort profile offers detailed information about the study’s background and the methodologies used in each sweep.^24^ The variables reported in this study were from the BCS70 sweeps at birth, age 5 and age 46.

### Patient and public involvement and engagement

A co-production workshop with 25 stakeholders informed the design of the research.^25^ The stakeholders included people with policy or practice experience of the early-life course from backgrounds including integrated care boards, healthcare practitioners, academics, council employees and not-for-profit organisations. Through this workshop that was co-produced with four public contributors, stakeholders identified age 5 as an important transitional timepoint, with the start of primary school representing a feasible and practical time point for interventions to improve long-term health. As such, this informed our decision to focus on this age point for this research.

### Outcome measurement

The outcome was self-reported MLTCs and role limitation at age 46. MLTCs was defined as the reporting of two or more LTCs from a list of all 22 conditions reported including: asthma, diabetes, cancer, high blood pressure, heart problems, eczema, chronic fatigue syndrome, stomach, bowel or gall bladder conditions, bladder or kidney conditions, liver disease, arthritis, stroke, depression, anxiety, hearing loss in one or both ears, epilepsy, eye conditions—blindness and low vision, eye condition—diabetes associated disease, eye condition—glaucoma, endometriosis, Meniere’s disease and psoriasis. We were limited by the specific conditions included by those who conducted the age 46 data sweep. As a result, several common conditions were not available for analysis, including, but not limited to, chronic obstructive pulmonary disease, irritable bowel syndrome, chronic pain, autism and osteoporosis. Further we acknowledge that by using broader groupings of conditions, such as stomach, bowel or gall bladder conditions, or bladder or kidney conditions, has meant there is the possibility some conditions could have been misclassified.

Role limitation was assessed using responses from the 36-Item Short Form Survey.^26^ Limitations due to physical health were measured using four binary (0/1) items. Lower average scores indicated greater functional impairment caused by physical health issues during the 4 weeks preceding the interview. Scores were calculated as the mean of the items answered. In line with previous guidance,^27^ a response of 0 (‘yes’) signified that physical health problems had limited the participant. Similarly, role limitation due to emotional problems was evaluated using three binary (0/1) items. Again, lower average scores reflected greater functional impairment due to emotional issues in the same 4-week period. A score of 0 (‘yes’) indicated that emotional health had limited the participant, following the same guidance.^27^ The term ‘emotional problems’ is derived from the original 36-Item Short Form Survey^26^ and refers to mental health.^28^ The specific survey items used to assess both types of role limitation are listed in online supplemental materials 1.

We created a binary variable indicating the presence of MLTCs with role limitation by grouping individuals who reported role limitations due to either physical or emotional health problems and who also reported MLTCs. This approach required the assumption that the reported role limitations were attributable to the LTCs under consideration. The comparison group in all the models comprised individuals with neither role limitations nor MLTCs. In subsequent analysis we additionally explored those who had the outcome of MLTCs regardless of role limitation status, and the comparison group was those who reported no MLTCs regardless of role limitation status.

### Analytical sample

From the 8581 participants who responded at the age 46 sweep, the sample was reduced because the analysis required cohort members to have also participated in the age-5 and birth sweeps, ensuring availability of prospectively collected early childhood data. We then applied a complete-case approach, restricting the analytic sample to individuals with no item-level missingness across any of the predictor variables and no missing data on the outcome. Finally, in accordance with the study design, the comparison group comprised only participants with neither role limitations nor MLTCs at age 46. These sequential restrictions resulted in a final analytical sample of 5007 participants.

### Selection of candidate predictors

Potential candidate predictors were selected from previous work that conceptually identified 12 domains of early-life factors as being important for MLTC risk^22^ and explored how potential multiple early-life determinants of MLTC can be characterised across three UK cohort studies.^29^ In this work, we identified potential candidate predictors recorded at birth and age 5 that mapped onto these 12 early-life domains, and included all relevant variables to try represent as many of these early-life domains as possible.

### Candidate predictors at birth

Seven variables at birth were identified: breastfeeding, maternal smoking during pregnancy, parity, gestational age, birth weight, teenage pregnancy and maternal age.

### Candidate predictors at age 5

33 variables were identified at age 5, five variables were dropped during complete case analysis due to high percentage of missing data (missing above 20%) resulting in 28 variables at age 5. These were: child’s sex, number of persons in the household, mother’s age, father’s age, mother figure’s relationship to child, separations between parents and child within 12 months of birth, breastfeeding, number of immunisations, mother and child separated >1 month, child seen by school medic <4 years old, bronchitis, hearing difficulty, eczema, wheezing, mother’s employment, mother depressed and a range of opinion variables including: ‘children should not talk at the table’, ‘children should accept what their parents say’, ‘parents should treat their children as equals’, ‘unquestioning obedience is not a good thing’, ‘people who are satisfied should not struggle for more’, ‘children should not talk back to their parents’, ‘children must do things without explanation’, ‘children must be themselves’, ‘children do not understand others feelings’, ‘a good child is one that does not have to be told the same thing more than once’. These opinion variables were selected because they mapped onto the parental-family domain, which was identified as being important for future MLTC risk in earlier work.^22 29^ They were selected to give a holistic view of the environment the child grew up in, as they provided an overview of parental attitudes, parenting behaviours and parenting styles. Other variables included Rutter behaviour scale an established scale to measure signs of behaviour disorders in children and teenagers^30 31^ and the maternal Malaise index—a set of self-completion questions which combine to measure levels of psychological distress, or depression.^32^

### Statistical analysis

Statistical analysis was performed using Stata V.18. Backwards stepwise elimination was used to select early-life variables to be included in the models. This automatic selection procedure started with the full model (including all candidate predictor variables) and sequentially removed variables based on a series of hypothesis tests. Variables were removed sequentially based on their contribution to model fit, using a threshold of 0.157. This threshold was selected as it corresponds to the selection criterion implied by the Akaike information criterion (AIC), as described by Atkinson^33^ and further discussed by Heinze *et al*.^34^ Using this slightly higher threshold reduces the risk of overfitting by allowing potentially informative predictors to remain in the model during the selection process, thereby providing a more conservative approach than relying on conventional significance levels (ie, 0.05 or 0.01). We then mapped the variables retained in the model to the 12 conceptual domains of early-life risk factors for future health outcomes.^22^

Four models explored the relationship between retained variables following stepwise backwards elimination and two outcomes. Based on this logistic regression modelling and using the ‘predict’ function in STATA,^35^ predicted risk scores for each cohort member for each of the outcomes were calculated. The four prediction models were:

1. **A5-RL:** Age 5 predictors only and MLTC with role limitation.
2. **A5-NoRL:** Age 5 predictors only and MLTC without role limitation.
3. **A5B-RL:** Age 5 and birth predictors and MLTC with role limitation.
4. **A5B-NoRL:** Age 5 and birth predictors and MLTC without role limitation.

Model performances were assessed using discrimination. Discrimination is a measure of how well the model differentiates between the individuals. The area under the curve (AUC) was used to summarise the overall discriminatory ability of the models. The AUC was classified as 0.6–0.7 poor, 0.7–0.8 fair, 0.8–0.9 good and 0.9–1.0 excellent.^36^

## Results

736, or 11.9%, of the sample had MLTCs with role limitation at age 46. Table 1 presents the distribution of all predictor variables at age 5 and at birth by MLTC with role limitations at age 46. Girls were notably over-represented in the MLTC with role limitations group compared with boys (15.1% vs 8.6%), and higher proportions of children with household size equal to or more than five reported the outcome compared with household size four or less (13.5% vs 10.7%). Several childhood health conditions showed consistent over-representation (vs no condition): wheezing (15.5% vs 11.0%), bronchitis (15.7% vs 11.1%), hearing difficulties (13.7% vs 11.7%), eczema (12.6% vs 11.7%) and three or fewer immunisations (14.1% vs 10.9%). 15.3% of children whose mothers reported depression at age 5 developed the outcome compared with 10.2% whose mothers did not. Several parental opinion variables (used as proxies for the psychosocial family environment) showed strong over-representation, such as stronger endorsement that children ‘should not talk at the table’ (16.3% vs 9.5%) or that ‘unquestioning obedience is not a good thing’ (15.7% vs 10.7%) among those with MLTCs and role limitation. Birth-related factors also demonstrated differences. Children whose mothers smoked during pregnancy (14.9% vs 10.1% in non-smokers) and who were born to mothers who had previously had a teenage pregnancy (16.3% vs 10.4%) were more likely to develop the outcome.

**Table 1:** Summary of predictor variables and outcome for the sample inputted into the models

	No MLTC or role limitation (%)	MLTC with role limitations (%)
**Age 5**		
Child’s sex		
Boy	2346 (91.4)	220 (8.6)
Girl	2124 (84.9)	377 (15.1)
Number of persons in household		
4 or less	2729 (89.3)	326 (10.7)
5 or more	1741 (86.5)	271 (13.5)
Mother’s age	26.0±5.9	26.0±6.1
Father’s age	27.9±8.5	27.2±8.7
Mother figure’s relationship to child		
Natural mother	4415 (88.3)	583 (11.7)
Non-natural mother	56 (80.0)	14 (20.0)
Separated immediately after birth (within 1 month)		
None	4167 (88.5)	542 (11.5)
Separated	197 (86.93)	32 (14.0)
Breastfeeding*		
More than 3 months	577 (90.9)	58 (9.1)
1–3 months	490 (88.8)	62 (11.2)
Up to a month	722 (87.7)	101 (12.3)
Never breastfed	2644 (87.7)	371 (12.3)
Number of immunisations		
4 or more immunisations	3487 (89.1)	428 (10.9)
3 or less immunisations	880 (85.9)	144 (14.1)
Mother and child separated >1 month		
No	4298 (88.4)	567 (11.7)
Yes	173 (85.2)	30 (14.8)
Child seen by school medic <4 years old		
No	2661 (88.5)	347 (11.5)
Yes	1595 (87.9)	219 (12.1)
Bronchitis		
Never had bronchitis	3644 (88.9)	457 (11.1)
Had/has bronchitis	652 (84.4)	121 (15.7)
Hearing difficulty		
Never had hearing difficulty	3928 (88.3)	520 (11.7)
Had/has hearing difficulty	372 (86.3)	59 (13.7)
Eczema		
Never had eczema	3781 (88.3)	503 (11.7)
Had/has eczema	500 (87.4)	72 (12.6)
Wheezing–ever occurred		
No	3604 (89.1)	442 (11.0)
Yes	825 (84.5)	151 (15.5)
Mothers’ employment		
Full-time housewife	2495 (88.3)	332 (11.7)
Regular work away	1468 (89.0)	181 (11.0)
Occasional work away	138 (82.14)	30 (17.9)
Regular home work	225 (87.6)	32 (12.5)
Occasional home work	58 (90.6)	6 (9.4)
Has two jobs	21 (84.0)	4 (16.0)
Mother depressed		
No	3135 (89.8)	356 (10.2)
Yes	1269 (84.7)	229 (15.3)
Children should not talk at the table		
Strongly agree	324 (83.7)	63 (16.3)
Mildly agree	1016 (86.8)	155 (13.2)
Cannot say	71 (85.5)	12 (14.5)
Mildly disagree	1360 (87.9)	187 (12.1)
Strongly disagree	1661 (90.5)	174 (9.5)
Children should accept what their parents say		
Strongly agree	1115 (87.9)	154 (12.1)
Mildly agree	1223 (87.9)	168 (12.1)
Cannot say	218 (88.6)	28 (11.4)
Mildly disagree	1108 (88.3)	147 (11.7)
Strongly disagree	761 (89.0)	94 (11.0)
Parents should treat their children as equals		
Strongly agree	867 (90.1)	95 (9.9)
Mildly agree	1300 (87.6)	184 (12.4)
Cannot say	244 (89.1)	30 (11.0)
Mildly disagree	1174 (87.7)	164 (12.3)
Strongly disagree	832 (87.7)	117 (12.3)
Unquestioning obedience is not a good thing		
Strongly agree	1391 (89.3)	167 (10.7)
Mildly agree	1681 (89.0)	208 (11.0)
Cannot say	307 (84.3)	57 (15.7)
Mildly disagree	680 (85.2)	118 (14.8)
Strongly disagree	339 (90.4)	36 (9.6)
People who are satisfied should not struggle for more		
Strongly agree	413 (86.0)	67 (14.0)
Mildly agree	593 (88.1)	80 (11.9)
Cannot say	217 (90.8)	22 (9.2)
Mildly disagree	1201 (87.9)	166 (12.1)
Strongly disagree	2001 (88.7)	255 (11.3)
Children should not talk back to their parents		
Strongly agree	1086 (88.6)	140 (11.4)
Mildly agree	1440 (88.5)	188 (11.6)
Cannot say	164 (84.1)	31 (15.9)
Mildly disagree	1254 (88.2)	168 (11.8)
Strongly disagree	476 (88.2)	64 (11.9)
Children must do things without explanation		
Strongly agree	478 (84.3)	89 (15.7)
Mildly agree	1174 (88.3)	155 (11.7)
Cannot say	329 (87.5)	47 (12.5)
Mildly disagree	1170 (88.7)	149 (11.3)
Strongly disagree	1235 (89.2)	150 (10.8)
Children must be themselves		
Strongly agree	464 (87.9)	64 (12.1)
Mildly agree	1461 (88.1)	198 (12.0)
Cannot say	346 (90.6)	36 (9.4)
Mildly disagree	1412 (88.6)	182 (11.4)
Strongly disagree	734 (87.3)	107 (12.7)
Children do not understand others’ feelings		
Strongly agree	1146 (88.4)	151 (11.6)
Mildly agree	1318 (87.5)	189 (12.5)
Cannot say	152 (88.9)	19 (11.1)
Mildly disagree	1218 (89.1)	149 (10.9
Strongly disagree	582 (87.4)	84 (12.6)
A good child is one that does not have to be told the same thing more than once		
Strongly agree	552 (87.5)	79 (12.5)
Mildly agree	1039 (89.3)	124 (10.7)
Cannot say	177 (87.2)	26 (12.8)
Mildly disagree	1547 (87.2)	227 (12.8)
Strongly disagree	1108 (89.2)	134 (10.8)
Rutter behaviour scale	9.0±5.3	10.5±6.1
Maternal Malaise index	1.1±0.6	1.2±0.7
**Birth**		
Breastfeeding*		
More than 3 months	577 (90.9)	58 (9.1)
One month to 3 months	490 (88.8)	62 (11.2)
Up to a month	722 (87.7)	101 (12.3)
Never breastfed	2644 (87.7)	371 (12.3)
Maternal smoking		
Non-smoker	2279 (89.9)	256 (10.1)
Stopped pre-pregnancy	669 (91.3)	64 (8.7)
Stopped during pregnancy	239 (89.5)	28 (10.5)
Smoked during pregnancy	1841 (85.2)	321 (14.9)
Gestational age (in days)	260.5±48.9	270.8±56.6
Parity		
0	2020 (89.3)	242 (10.7)
1	1740 (89.0)	215 (11.0)
2	756 (86.0)	123 (14.0)
3	308 (85.3)	53 (14.7)
4	137 (86.7)	21 (13.3)
5 or more	102 (84.3)	19 (15.7)
Birthweight (grams)	3533±480.7	3326.0±517.7
If mother was ever a teen mum		
No	4385 (89.6)	456 (10.4)
Yes	1319 (83.7)	215 (16.3)
Maternal age	24.5±4.6	26.0±5.3

* Breastfeeding as a predictor variable was identified in both the age 5 and birth sweeps.

MLTC, multiple long-term condition.

As demonstrated by table 2 across the four models, a total of 21 unique predictors were retained. Two variables, Rutter behaviour and sex, were consistently included in all models, suggesting their robustness as predictors across different outcome specifications. Five variables were retained in only one model, including hearing difficulties (A5B-NoRL), teenage pregnancy (A5B-NoRL), school medic visit before age 4 (A5B-RL), maternal smoking (A5B-RL) and parity (A5B-RL), indicating more limited relevance or specificity to particular outcomes.

**Table 2:** List of predictors retained for each of the four models, the number of predictors retained for each variable and the AUC of each model

	Model 1 (A5-RL)		Model 2 (A5-NoRL)		Model 3 (A5B-RL)		Model 4 (A5B-NoRL)	
	N=5007		N=5007		N=4802		N=4802	
	Predictor: age 5		Predictor: age 5		Predictors: age 5 and birth		Predictors: age 5 and birth	
	Outcome: MLTCs and role limitation		Outcome: MLTCs		Outcome: MLTCs and role limitation		Outcome: MLTCs	
Number of predictors retained	7		10		12		11	
Predictors:	Coefficient	95% CI	Coefficient	95% CI	Coefficient	95% CI	Coefficient	95% CI
Number of immunisations	0.16	−0.5 to 0.4	–	–	–	–	–	–
Wheezing diagnosis	0.27	0.33 to 0.5	0.28	0.13 to 0.43	0.23	−0.01 to 0.48	0.29	0.14 to 0.44
Child’s sex	0.45	0.24 to 0.66	0.53	0.40 to 0.65	0.45	0.23 to 0.66	0.53	0.40 to 0.65
Rutter summed	0.33	0.14 to 0.52	0.2	0.008 to 0.310	0.02	0.01 to 0.05	0.02	0.006 to 0.030
Maternal malaise grouped	0.33	0.10 to 0.55	–	–	0.26	0.03 to 0.49	–	–
Eczema	–	–	0.22	0.05 to 0.40	–	–	0.22	0.04 to 0.40
Mother depressed	–	–	0.21	0.07 to 0.34	–	–	0.16	0.02 to 0.30
Hearing difficulty	–	–	–	–	0.25	−0.08 to 0.60	0.19	−0.02 to 0.40
Child seen by school medic <4 years old	–	–	–	–	−0.8	−0.40 to 0.04	–	–
Gestational age (in days)	–	–	–	–	0.0009	0.003 to 0.0008	–	–
If mother was ever a teen mum	–	–	–	–	0.45	0.21 to 0.69	0.18	0.02 to 0.33
Separations between parents and child within 12 months of birth	–	–	–	–	–	–	−0.87	−1.73 to −0.01
Maternal smoking								
Non-smoker					Ref			
Stopped prepregnancy	–	–	–	–	−0.12	−0.47 to 0.23	–	–
Stopped during pregnancy					0.06	−0.45 to 0.56		
Smoked during pregnancy					0.22	−0.01 to 0.45		
Parity								
0					Ref			
1					0.16	−0.09 to 0.42		
2	–	–	–	–	0.29	−0.01 to 0.61	–	–
3					0.45	0.03 to 0.87		
4					0.4	−0.22 to 1.00		
5 or more					0.62	−0.08 to 1.33		
Mother’s employment								
Full-time housewife			Ref					
Regular work away			−0.33	−2.05 to 1.38				
Occasional work away			−0.06	−1.8 to 1.68	–	–	–	–
Regular home work	–	–	−0.31	−2.04 to 1.43				
Occasional home work			−0.07	−1.85 to 1.71				
Has two jobs			−0.57	−1.28 to 2.42				
Opinion: Parent’s should treat their child as equals								
Strongly agree	Ref		Ref		Ref		Ref	
Mildly agree	0.4	0.76 to 0.72	−0.11	−0.36 to 0.14	0.45	0.12 to 0.78	0.19	0.0 to 0.38
Cannot say	0.13	−0.53 to 0.56	0.11	−0.37 to 0.59	−0.09	−0.68 to 0.49	0.07	−0.24 to 0.38
Mildly disagree	0.49	0.17 to 0.81	−0.24	−0.49 to 0.12	0.51	0.18 to 0.85	0.28	0.08 to 0.47
Strongly disagree	0.37	0.03 to 0.72	−0.29	−0.54 to −0.03	0.34	−0.03 to 0.70	0.26	0.06 to 0.47
Opinion: Child should have unquestioning obedience towards their parents								
Strongly agree	Ref		Ref		Ref			
Mildly agree	0.2	−0.6 to 0.46	0.06	−0.9 to 0.21	0.18	0.08 to 0.45		
Cannot say	0.5	0.12 to 0.88	0.31	0.07 to 0.55	0.4	−0.01 to 0.80		
Mildly disagree	0.48	0.18 to 0.78	0.1	0.09 to 0.29	0.48	0.17 to 0.79	–	–
Strongly disagree	0.07	−0.37 to 0.50	0.006	−0.24 to 0.26	−0.004	−0.46 to 0.45		
Opinion: Child does not understand other’s feelings								
Strongly agree			Ref					
Mildly agree	–	–	−0.04	−0.21 to 0.12	–	–	–	–
Cannot say			0.15	−0.19 to 0.48				
Mildly disagree			0.11	−0.07 to 0.28				
Strongly disagree			0.21	−0.22 to 0.43				
Opinion: Children should not talk back at their parents								
Strongly agree			Ref				Ref	
Mildly agree	–	–	−0.44	−0.21 to 0.12	–	–	−0.02	−0.19 to 0.15
Cannot say			0.15	−0.19 to 0.48			0.21	−0.13 to 0.55
Mildly disagree			0.11	−0.69 to 0.28			0.12	−0.06 to 0.30
Strongly disagree			0.21	−0.022 to 0.43			0.21	−0.02 to 0.44
Opinion: Children should not talk at the table								
Strongly agree							Ref	
Mildly agree	–	–	–	–	–	–	−0.02	−0.19 to 0.15
Cannot say							0.21	−0.13 to 0.55
Mildly disagree							0.12	−0.06 to 0.30
Strongly disagree							0.21	−0.02 to 0.44
AUC	0.635 (0.606 to 0.662)		0.612 (0.600 to 0.633)		0.660 (0.632 to 0.689)		0.611 (0.593 to 0.628)	

Regression coefficients should be interpreted as indicators of each variable’s contribution to the prediction model rather than as measures of association.

AUC, area under the curve; MLTC, multiple long-term condition.

As indicated in table 2 model performance, as measured by the (AUC), ranged from 0.61 to 0.66. Model 3 (A5B-RL) achieved the highest predictive accuracy (AUC=0.66), while models 2 (A5-NoRL) and 4 (A5B-NoRL) showed the lowest performance (AUC=0.61). Although the number of retained variables increased from model 1 (A5-RL) (7 variables) to model 3 (A5B-RL) (12 variables), this did not consistently translate into improved predictive performance.

Table 3 demonstrates how the retained variables mapped to seven conceptual domains: developmental attributes, transgenerational factors, childhood health, demographics, socioeconomic factors, parental-family factors and prenatal/antenatal/birth factors. In Model 1 (A5-RL), the seven variables retained included Rutter behaviour, which was mapped to the domain of developmental attributes, and the maternal malaise index, representing the transgenerational factors domain. The childhood health domain was represented by the number of immunisations and wheezing diagnoses, while sex was included under the demographics domain. The socioeconomic factors domain was captured through the father’s occupation. Finally, the parental-family factor domain was represented by two parental opinions: that children should be treated as equals and that they should show unquestioning obedience to their parents.

**Table 3:** Predictors retained for each model and the early-life domain they represent

Domain	Variable	Model 1 (A5-RL) Predictors: age 5 Outcome: Multiple long-term conditions with role limitations	Model 2 (A5-NoRL) Predictors: age 5 Outcome: Multiple long-term conditions	Model 3 (A5B-RL) Predictors: age 5 and birth Outcome: Multiple long-term conditions with role limitations	Model 4 (A5B-NoRL) Predictors: age 5 and birth Outcome: Multiple long-term conditions
Developmental attributes	Rutter behaviour	Yes	–	Yes	Yes
Transgenerational	Maternal Malaise Index	Yes	–	Yes	–
	Mother’s depression diagnosis	–	Yes	–	Yes
Childhood health	Number of immunisations	Yes	Yes	Yes	–
	Wheezing diagnosis	Yes	Yes	Yes	Yes
	Eczema diagnosis	–	Yes	–	Yes
	Child seen by school medic before age 4	–	–	Yes	–
	Hearing difficulty	–	–	–	Yes
Demographic	Sex	Yes	Yes	Yes	Yes
Socioeconomic	Father’s occupational	Yes	–	–	–
	Mother’s occupation	–	Yes	–	–
Parental-family factors	Parents should treat their child as equals	Yes	–	Yes	Yes
	Children should have unquestioning obedience to parents	Yes	–	Yes	–
	People should be satisfied with what they have and not ask for more	–	Yes	–	–
	Children should not talk back at their parents	–	–	–	Yes
	Children should not talk at the table	–	–	–	Yes
Prenatal, antenatal, neonatal and birth	Maternal smoking	–	–	Yes	–
	Gestational age	–	–	Yes	–
	Parity	–	–	Yes	–
	Ever had a teenage pregnancy	–	–	Yes	Yes
Adverse childhood experience	Parental separation after birth	–	Yes	–	Yes

Model 2 (A5-NoRL) retained 10 variables spanning a broader range of domains. Separation after birth was included as an indicator of the adverse childhood experiences domain (ACE). The childhood health domain was represented by the number of immunisations, wheezing diagnosis and eczema diagnosis. Rutter behaviour was again included under the developmental attributes domain, while maternal depression diagnosis was mapped to the transgenerational factors domain. The socioeconomic domain was represented by the mother’s occupation, and sex was included under the demographic domain. The parental-family factors domain was captured by the opinion that people should be ‘satisfied with what they have and not ask for more’.

Model 3 (A5B-RL) incorporated 12 variables, offering the most comprehensive representation across domains. Rutter behaviour was again included under the developmental attributes domain, and the maternal malaise index represented the transgenerational factors domain. The childhood health domain was captured through the number of immunisations, wheezing diagnosis and whether the child had been seen by a school medic before the age of four. The demographic domain was represented by sex. The parental-family factors domain included parental opinions on treating children as equals and on unquestioning obedience. Additionally, the prenatal, antenatal and birth factors domain was represented by maternal smoking, gestational age and parity.

Model 4 (A5B-NoRL) retained eleven variables, spanning seven conceptual domains. Rutter behaviour was included under the developmental attributes domain, while the ACE domain was represented by separation after birth. The childhood health domain included wheezing, eczema and hearing difficulties. The transgenerational factors domain was captured through the mother’s depression diagnosis, and the demographic domain was represented by sex. The parental-family factors domain included three parental opinions: that children should be treated as equals, that children should not talk at the table and that children should not talk back to their parents. Finally, the prenatal, antenatal and birth domain was represented by whether the individual had ever experienced a teenage pregnancy.

In online supplemental materials 2, we applied both ridge and lasso regularisation to our modelling. This yielded results that were highly comparable to those presented in the manuscript. Overall, these findings demonstrated that alternative regularisation approaches do not materially alter the predictive performance of our models.

## Discussion

Using early-life factors considered at birth and age 5 to predict the risk of MLTCs with role limitation at age 46 yielded poor discrimination in all models tested. Model 3 (A5B-RL) achieved the highest AUC of 0.66, whereas the lowest, model 2 (A5-NoRL), was 0.61. The AUC scores marginally improved when birth predictors were included in the model yet only yielded small improvement in discrimination. The models had a higher AUC when the outcome included role limitation (models 1 (A5-RL) and 3 (A5B-RL)) compared with MLTC only (models 2 (A5-NoRL) and 4 (A5B-NoRL)). Given the poor performance across all models, the findings suggest that childhood factors measured at birth and age 5 may not be sufficient for accurately predicting MLTCs, either with or without role limitations. This points to two possibilities: first, that additional exposures later in the life course may play a more substantial role in predicting MLTCs; and second, that important early-life factors may exist but were not captured in the available data.

Retained predictors covered a wide span of domains. Several of these predictors and their respective domains were expected, supporting previous limited research on examining the association between MLTC and socioeconomic factors, as well as the limited evidence regarding other behavioural determinants of MLTCs in childhood. The retained socioeconomic and demographic factors such as the child’s sex and parents’ job occupation alongside childhood health factors such as hearing problems, have previously been linked to MLTC.^37^,^39^ Several antenatal, prenatal, neonatal and birth predictors were retained, such as gestation, parity, teenage pregnancy and maternal smoking, further emphasising the role of birth factors in later-life MLTC. Both hearing problems and childhood asthma have been linked to birth factors such as premature gestation and low birth weight, further providing more insight into the pathway of developing MLTC.^40 41^

Many of the variables retained across all four models were parental opinion variables, drawn from the parental-family factors domain. These were included as proxies to capture aspects of the family environment in which the child was raised. Notably, such parental opinion data are not routinely collected and are rarely considered in research on early-life determinants of later health outcomes, with even less attention given to their role in the development of MLTCs. Our finding suggests that there may be wider, under-recognised determinants of health in childhood that are often overlooked in epidemiological research. Parental attitudes and perceptions may act as proxies for broader psychosocial environments, which appear to enhance predictive performance even if their mechanisms of influence cannot be inferred from prediction models. These findings suggest that household factors might influence a child’s development and long-term health trajectory, shaping vulnerability to MLTCs over the life course. Recognising and incorporating such variables into predictive models is important, as it broadens our understanding of what constitutes meaningful early-life exposures. It also highlights the need for richer, more nuanced data collection in longitudinal studies to better capture the social and relational contexts of childhood. Doing so could improve the design of preventative interventions by identifying modifiable family-level factors that contribute to the onset and progression of MLTCs.

Given the significant healthcare and economic burden of MLTC in the UK and globally, this research looked to identify potential important predictors of MLTC that could then inform prevention modelling in future research and potentially look to reduce pressure on healthcare systems. In the UK, this is especially important, where it is estimated that more than half of the population aged 65 and above suffers from two or more LTCs, and it is predicted that by 2035, two-thirds of people aged over 65 will experience MLTC. The aims of this research support the wider shift across the UK health system towards ill health prevention and addressing health inequities.^42^,^44^

Although all four prediction models demonstrated poor discrimination, the findings remain relevant for public health. Across the UK, there has been a strategic shift towards prevention-focused health research, demonstrated by recent policy documents from the Department of Health and Social Care^45^ and Public Health Scotland,^46^ both of which emphasise prevention and the wider determinants of health. Research that evaluates the potential predictive value of early-life factors is therefore important, even when predictive accuracy is modest, as it helps inform where prevention efforts may or may not be most appropriately targeted. Identifying the limitations of prediction models contributes to a transparent understanding of the boundaries of long-term prediction and reduces the risk of overinterpreting early life indicators. Our findings also reinforce that mid-life MLTC risk is unlikely to be meaningfully predicted using only the information available at birth or age 5 we have considered in this paper. This highlights that later developmental periods such as adolescence or early adulthood may provide additional informative opportunities for prediction modelling and preventative action. It is also important to acknowledge that there may have been other relevant variables at age 5 that were not available within this cohort and therefore could not be considered. Finally, the importance of parental attitude variables, which are often unavailable in alternative datasets such as electronic healthcare records, offers additional insight. Our results suggest that aspects of the family and psychosocial environment could offer predictive value in MLTC modelling and provide support for the expansion of data collection focusing on the wider determinants of health.

We acknowledge that our results are representative only of this specific cohort and cannot be extrapolated directly to other cohorts or to the general population. Social norms and behaviours in the 1970s, when cohort members were aged 5, differ in several important ways from current patterns. For example, two of our retained predictors included parental separation after birth and maternal smoking. Maternal smoking prevalence is considerably lower in contemporary populations than it was in the 1970s,^46^ whereas parental separation is now more common.^47^ As such, caution should be taken when interpreting these findings in relation to other cohorts.

However, many of the underlying mechanisms linking our retained predictors to later health, such as socioeconomic disadvantage, early childhood health, family environment, maternal mental health and developmental attributes, are likely to remain relevant across cohorts. Supporting this, the life course theories discussed in the introduction conceptualise early-life influences as structural rather than time bound, and several of the early-life domains included in our prediction models align with these broader structural determinants of health.

### Strengths and limitations

This study drew on a large, nationally representative birth cohort that provides some of the most comprehensive early-life data available in Britain. The cohort includes detailed biological, social, environmental, behavioural and familial information across multiple early-life domains that have been conceptualised to influence the development of LTCs in adulthood.^22^ Such rich data would not typically be available in other sources, such as electronic health records in either primary or secondary care. The prospective, longitudinal design, with data collected across multiple sweeps from birth through adulthood, minimised recall bias and helped establish clear temporal ordering between predictors and the outcome, enabling a robust examination of how early-life factors predict midlife MLTC. The breadth of these early-life measures also allowed us to systematically assess the predictive contribution of numerous early-life domains previously conceptualised to influence long-term health trajectories,^22^ but not formally evaluated within prediction models.

However, the cohort is representative of births occurring in Britain in 1970, which was predominantly a White British population, and therefore our results lack ethnic diversity. Further, the analysis was restricted to participants who were present in all three selected sweeps of the BCS70, resulting in a sample of approximately 5000 individuals from the original cohort. This introduces the potential for selection bias, as individuals who remain in long-term cohort studies are often systematically different from those who are lost to follow-up. Specifically, people with poorer health, lower socioeconomic status or more unstable life circumstances are more likely to be lost to follow-up.

To examine this further, we compared participants included in our complete case sample at age 46 with those who were missing outcome data. Consistent with previous work on this cohort, we found similar patterns of attrition: men were slightly more likely to be lost to follow-up than women (62% vs 60%); participants with lower parental qualifications had higher attrition (67% with no qualifications vs 58% with GCSE or above); children who had seen a school medical doctor by age 5 (a proxy for early childhood health) were marginally more likely to be lost to follow-up (62% vs 61%); those who had been separated from their mother during childhood were more likely to be lost to follow-up (69% vs 61%); and children whose mothers self-reported depression at age 5 were also more likely to be lost to follow-up (65% vs 59%). These patterns reinforce the possibility of selection bias and suggest that the prevalence of MLTCs in our analytical sample may be underestimated.

The data for the candidate predictors and MLTC and role limitation outcomes were all self-reported. Only a small number of MLTCs were captured with some common conditions not considered. Additionally, the concept of role limitation captures only one dimension of the burden experienced by individuals living with MLTCs. For example, Holland *et al*^2^ identified eight distinct themes of work-related burden among people with MLTCs, suggesting that the lived experience of these conditions is multifaceted. Future research could therefore benefit from exploring a broader range of burden indicators (where data allows). The small sample sizes in candidate predictors with the outcome precluded the opportunity for subgroup analysis. Therefore, it was not possible to look at early-life predictors for certain clusters of conditions for burden, which is of value, given some LTCs may be more burdensome than others.

Further, some candidate predictors were removed due to high levels of missing data, and there were many duplicate candidate predictors that appeared in both the age 5 and birth datasets, such as parity, gestation and breastfeeding. Several childhood domains were not captured in the age 5 dataset, either because data was unavailable or were excluded due to high levels of missing data. These included the child’s health behaviours such as sleeping difficulty, daytime wetting and overeating, as well as education and health literacy, such as school attendance which has been found to be important for adult health outcomes such as health service use.^48^ If domains were captured, such as ACE, demographics and the transgenerational impact of parent health, they were only represented by a few predictors.

Finally, we note that using backward stepwise elimination carries recognised limitations, including the potential for model instability and an increased risk of overfitting.^49 50^ Stepwise approaches have also been shown to produce biased coefficient estimates.^51^ These concerns are enhanced when research involves rare outcomes, where greater sampling variability can make variable selection more unreliable. Consequently, the results should be interpreted with caution. However, as discussed by Royston and Sauerbrei,^52^ although all variable selection strategies attract criticism, backward elimination offers several practical advantages. Because it begins with the full model, it tends to retain strong predictors, and using a threshold such as p=0.157 provides a good approximation to AIC-based selection. It has also generally been found to perform better than forward selection procedures, as forward methods allow variables to enter the model only once and can miss important combinations of predictors.^52^

## Conclusions

Using early-life factors measured at birth and age 5 to predict the risk of MLTCs with role limitation at age 46 yielded poor discrimination. While the early-life course may be important for the prevention or delay of MLTCs in adulthood, it may not be sufficient on its own. There may be a need to incorporate additional exposures later in life or to consider other early-life factors that were not captured in the available data. Further exploration of predictors across different childhood stages is recommended to strengthen and inform the design of preventative interventions targeting MLTCs and associated role limitations.

## Supplementary material

10.1136/bmjph-2025-004443 online supplemental file 1

10.1136/bmjph-2025-004443 online supplemental file 2

## Data Availability

Data may be obtained from a third party and are not publicly available.
