# Supplementary material for: Predicting multiple long-term conditions with role limitation at age 46 using early-life data from the 1970 British Cohort Study
Source: BMJ Public Health. 2026 Jun 28;4(2):e004443. doi: 10.1136/bmjph-2025-004443 (PMC13331061; doi:10.1136/bmjph-2025-004443)
Supplement: online supplemental file 1 [file bmjph-4-2-s001.pdf]

Predicting Multiple Long-Term Conditions with Role Limitation at Age 46 using early life data from the 1970 British Cohort Study.

Supplementary Materials

Supplementary Materials 1. SF-36 role limitations variables

SF-36 Role-limitations due to physical health

Physical health score coded on 4 items. Lower scores indicate greater lifestyles limitations as a result of physical health problems in the four week prior to interview. All four items are coded the same. Responses of ‘Yes’ score 0, as health has limited the cohort member (1=0) while ‘No’ is 100 (2=100). Scores are the mean of the total number of questions answered (1-4 responses).

4 items:

1. Have you cut down the amount of time you spent on work or other activities
2. Have you accomplished less than you would like
3. Were limited in the kind of work or other activities
4. Had difficulty performing the work or other activities (for example, it took extra effort)

SF-36 Role-limitations due to emotional health

Physical health score coded on 3 items. Lower scores indicate greater lifestyles limitations as a result of emotional problems in the four week prior to interview. All three items are coded the same. Responses of ‘Yes’ score 0, as health has limited the cohort member (1=0) while ‘No’ is 100 (2=100). Scores are the mean of the total number of questions answered (1-3 responses).

3 items:

1. Have you cut down the amount of time you spent on work or other activities
2. Have you accomplished less than you would like
3. Didn’t do work or other activities as careful as usual
