# Supplementary material for: Predicting multiple long-term conditions with role limitation at age 46 using early-life data from the 1970 British Cohort Study
Source: BMJ Public Health. 2026 Jun 28;4(2):e004443. doi: 10.1136/bmjph-2025-004443 (PMC13331061; doi:10.1136/bmjph-2025-004443)
Supplement: online supplemental file 2 [file bmjph-4-2-s002.pdf]

Supplementary Materials 2. AUC for the four models under ridge and lasso regularisation

	**Model 1 (A5-RL)**<br>*N=5007*	**Model 2 (A5-NoRL)**<br>*N=5007*	**Model 3 (A5B-RL)**<br>*N=4802*	**Model 4 (A5B-NoRL)**<br>*N=4802*
	Predictor: Age 5<br>Outcome: MLTCs and role limitation	Predictor: Age 5<br>Outcome: MLTCs	Predictors: Age 5 and birth<br>Outcome: MLTCs and role limitation	Predictors: Age 5 and birth<br>Outcome: MLTCs
**AUC**	0.635 (0.606 – 0.662)	0.612 (0.600 – 0.633)	0.660 (0.632 – 0.689)	0.611 (0.593 – 0.628)
**AUC – ridge regularisation**	0.636 (0.607 – 0.664)	0.610 (0.600 – 0.627)	0.654 (0.625 – 0.682)	0.615 (0.598 – 0.632)
**AUC – lasso regularisation**	0.635 (0.607 – 0.664)	0.610 (0.593 – 0.626)	0.650 (0.620 – 0.678)	0.614 (0.597 – 0.632)
